# The Effect of Modeling on Self-Efficacy and Flow State of Adolescent Athletes Through Role Models

**DOI:** 10.3389/fpsyg.2021.661557

**Published:** 2021-06-14

**Authors:** Seungjoo Lee, Sungho Kwon, Jihoon Ahn

**Affiliations:** Department of Physical Education, Seoul National University, Seoul, South Korea

**Keywords:** role model, modeling, self-efficacy, flow state, adolescent athlete

## Abstract

This study aimed to verify the effects of role modeling on adolescent athletes’ self-efficacy and flow state. The subjects were middle school and high school athletes registered with the Korean Sport & Olympic Committee. From the collected data, descriptive statistics, confirmatory factor analysis, correlation analysis, and structural equation model analysis were performed. To verify the mediating effects of self-efficacy in the relationship between modeling and flow state, structural equation modeling analysis was conducted. The direct effects of adolescent athlete modeling on flow state (*β* = 0.416, *B* = 0.244, *p* < 0.01) and self-efficacy (*β* = 0.479, *B* = 0.500, *p* < 0.01) were all significant, and the direct effects of self-efficacy on flow state (*β* = 0.404, *B* = 0.227, *p* < 0.01) were also significant. Furthermore, it was confirmed that the indirect effect of modeling on flow state (*β* = 0.194, *B* = 0.114, *p* < 0.01) was significant, and that the partial mediated effects of self-efficacy were significant. Thus, we confirmed that when adolescent athlete use modeling through a role model, their self-efficacy increased which in turn led to a positive effect on the ability to achieve a flow state.

## Introduction

It is human nature to emulate a person perceived to be superior to us. Having a “role model” is a spontaneous phenomenon. Most people at some point in their life are inspired by another human being and try to improve by modeling themselves after that person. Just as a toddler learns basic day-to-day skills like eating with a spoon by observing and imitating family members, adults can “mimic” their role models to imbibe their qualities. The impassioned goal of becoming like the role model can act as a motivating goal and challenge that can propel a person towards self-development. Role models subject to such imitation are defined as individuals who inspire individuals or groups of people or deserve to emulate (Ingall, 1997; Yancey, 1998).

In a survey of 1,000 ordinary men and women aged 18 or older conducted by Korean Research Survey (2020), 70% of all respondents replied they have a role model when asked, “Is there anyone who can be called a mentor or role model in your life?” In addition, each age group, from teens to 40s, was asked whether they had a role model. Among older adults, only 13.8% answered in the affirmative. In contrast, 78.3% of teenagers reported having a role model. Also, according to a survey divided into typical students and student athletes, 64.3% of typical students and 87.5% of student athletes reported having a role model they wanted to be like. It has been found that having a role model is beneficial for athletes as it motivates them to set goals and strive to achieve them (Morgenroth et al., 2015).

Examining prior studies related to role models, Hackett and Betz (1981) reported that the selection of a positive role model could have a positive effect on self-efficacy, creating expectations of competence in tasks that have never been tried before. Also, a study by Lockwood and Kunda (1999) found that positive role modeling strengthens a desire for success in a particular field, which in turn serves as a strong mental motivation for success. Role models also motivate and inspire new behaviors to set goals (Morgenroth et al., 2015). In addition, Huber (2013) reported four types of behaviors worth mimicking and noted that successful coaching is to allow young athletes to learn all acceptable behaviors by observing the person who exemplifies good behavior. Furthermore, Connolly (2017) explained the effects of positive role models in sports environments through observational learning and presented the importance and guidelines for the role of coaches in facilitating observation learning. The importance of role models can be seen through sports articles and interviews that discuss role modeling, in fact, many athletes try to boost self-efficacy or motivation and improve performance by observing and imitating the behavior of athletes they admire.

The function of these role models can be explained through the concept of vicarious experience presented by Bandura (1997). The vicarious experience can be easily understood as a way to enhance self-efficacy by observing the actions performed successfully by others. Vicarious experience helps reduce anxiety and leads to emulation of successful actions of role models (Schunk, 1989; Fitzsimmons et al., 1991). Such a vicarious experience can be called a form of modeling method. Modeling can be defined as learning from observation and copying other people’s actions by watching and listening to them and has been considered the most powerful means of communicating patterns of thought, behavior, values, and attitudes (Bandura, 1986). Looking at the prior studies related to modeling, Weiss et al. (1998) reported that modeling increased the self-efficacy of children with water phobia and had a positive effect on improving swimming skill. Furthermore, a study by Starek and McCullagh (1999) on adults showed improved self-efficacy through modeling and improved swimming skills. Also, a study by Law and Hall (2009) examined the effectiveness of modeling on novice sports participants and found that the subfactors of modeling in both group and individual events had a positive effect on self-efficacy. Bandura (1986) also stated that modeling is one of the main ways to improve self-efficacy. Self-efficacy refers to the degree of belief held by an individual regarding whether he or she can successfully perform a particular task, and it is a variable that has been found to have a positive relationship with athletic performance by a number of studies (Singh et al., 2009).

Meanwhile, in the field of sports psychology, peak performance is considered an important topic for athletes, coaches, and sports psychologists. Therefore, various studies have been conducted on approaches and mediation for producing peak performance in athletes (Kwon et al., 2018). These studies have verified many psychological variables that predict peak performance. In particular, the flow state was found to be a critical antecedent variable that predicted peak performance, and subsequently many studies were conducted on it (Jackson et al., 2001; Swann et al., 2012).

Flow refers to a state of mind and body that is automatic and controlled by the feeling of being completely absorbed in an action (Csikszentmihalyi, 1975) or refers to psychological state of losing oneself in a task and getting engrossed to the point of losing sense of time and space (Kwon, 2008). The flow state is important because it is closely related to the ultimate goal of athletes, peak performance. Flett (2015) reported that flow has a positive correlation with performance in tennis players. Also, flow is related to improving athletes’ performance (Pates et al., 2001) satisfaction with their life (Habe et al., 2019) and mental toughness (Crust and Swann, 2013). Furthermore, Csikszentmihalyi (1985) argued that the peak performance should be considered flow state. This can be explained as a mechanism by which athletes are totally absorbed in the competition situation and experience the peak performance through the expression of automated skill, which in turn proves that flow is a leading variable that can have a decisive impact on athletes’ peak performance (Jackson et al., 2001). Also, flow is closely related to the aforementioned self-efficacy of athletes.

Although no empirical studies have been reported on flow and self-efficacy, it has been reported that flow is related to sport-confidence, which is very similar to self-efficacy (Pineau et al., 2014), and has a positive correlation with both constructs (Vealey et al., 1998; Manzo et al., 2001). Feltz and Lirgg (2001) noted that the measurement of sport-confidence can be used as a measure of self-efficacy. In addition, Moritz et al. (2000) noted that self-efficacy is a variable that can be considered a specific sport-confidence depending on the situation. Furthermore, sport-confidence had a significant relationship (Kaufman et al., 2009) with flow and has been verified as an antecedent variable of flow state (Catley and Duda, 1997). In summing up these findings, it is believed that self-efficacy is related to flow and that improved self-efficacy through modeling can play a role in promoting the flow state.

It is suggested that having a positive role model improves the self-efficacy of being like them and has a positive impact on expectations of results and mental motivation. Furthermore, actively emulating a role model improves the self-efficacy of athletes and promotes the acquisition and performance of athletic skills. In addition, it was found that self-efficacy is related to the flow state. Through this process, we can infer that improved self-efficacy can serve as a variable to induce the flow state in athletes.

## Materials and Methods

### Participants

Currently, the Covid-19 infectious disease is spreading around the world, so the survey was conducted using the Google platform (online) to consider the safety of participants. The participants of this study set up the middle and high school athletes in Korea as a population. Thus, the athletic clubs registered in the Korean Sport & Olympic Committee (KSOC) were selected by using the purposive sampling. First, we delivered information about research recruitment to the coaches of each athletic club registered in KSOC. After that, a survey link was given to coaches in athletic clubs who wanted to participate. The survey was then conducted on adolescent athletes who received consent from their parents to participate in the study, and a total of 255 participant data were collected. If some of the survey responses were omitted or duplicated, the data were considered insufficient and 38 data were excluded. Therefore, the analysis was conducted from 187 participants (male: 146, female: 41). This study was approved by the Research Ethics Committee in advance of data collection. The participants were involved in the following sports: soccer (*n* = 77), judo (*n* = 4), weightlifting (*n* = 23), hockey (*n* = 8), fencing (*n* = 4), athletics (*n* = 45), taekwondo (*n* = 17), and cycling (*n* = 9).

### Measurements

#### Modeling

To measure the modeling of the adolescent athletes, we used the functions of observational learning questionnaire (FOLQ) developed by Cumming et al. (2005). First, we provided an explanation of the modeling concept so that adolescent athletes could understand and asked them to respond to the survey after recalling a role model that they admired or like. We modified the questionnaire to measure the level of modeling targeting at the role model (e.g., “I use modeling to understand how to perfectly perform a skill,” “I use modeling to learn how to cope with anxiety”). FOLQ consists of three factors (skill, strategy, performance), 17 questions, on a 7-point scale. Examples of the items in each factor include “I use observational learning through my role models to change how I perform a skill.” “I use observational learning through my role models make up new plans, strategies in my head.” and “I use observational learning through my role models to understand what it takes to be mentally tough.” If you want to know more about this items of questionnaire, see the Appendix 1. A confirmatory factor analysis was conducted to verify the construct validity of FOLQ. It was found that it met all the goodness-of-fit indices (*χ*^2^ = 361.218, TLI = 0.926, CFI = 0.937, RMSEA = 0.096). The Cronbach *α* of FOLQ was skill 0.939, strategy 0.937, and performance 0.936. The higher the score on this scale means more frequent use of the modeling method.

### Self-Efficacy

A self-efficacy questionnaire developed by Law and Hall (2009) was used. It consists of three factors (skill, strategy, performance) representing nine questions and a 10-point scale. Examples of the items in each factor include “I am confident I can learn the skills necessary to play this sport.” “I am confident I can learn the strategies necessary to play this sport.” and “I am confident I can stay focused when playing this sport.” If you want to know more about this items of questionnaire, see the Appendix 2. A confirmatory factor analysis was conducted to verify the construct validity of the self-efficacy questionnaire. It was shown to meet all the goodness-of-fit indices (*χ*^2^ = 67.250, TLI = 0.954, CFI = 0.970, RMSEA = 0.098). The internal consistency of the self-efficacy questionnaire (Cronbach α) was shown as skill 0.897, strategy 0.906, and performance 0.814. The higher the score on this scale means a higher level of self-efficacy.

### Flow State

The flow questionnaire developed by Kwon (2008) was used to measure the flow state of athletes. Before conducting the survey, we provided guidelines to ask them to respond to the survey after fully recalling a recent competition. The scale contains four subfactors (antecedent factor 3, threshold factor 4, experience factor 6, and consequence factor 5). In this study, the factors of threshold and experience were determined to be suitable for the purpose of this study to measure the flow state of athletes during the competition, so the scale was modified to 10 questions on two factors. Examples of the items in each factor include “I completely focus on the movement I’m doing.” and “I can lead the game as I want.” If you want to know more about this items of questionnaire, see the Appendix 3. A confirmatory factor analysis was conducted to verify the construct validity, following which we deleted items with factor loading of 0.5 or less. Therefore, the threshold factor was deleted. After deleting the seven items, it was found that all the goodness-of-fit indices were met (*χ*^2^ = 70.491, TLI = 0.937, CFI = 0.954, RMSEA = 0.096). The Cronbach *α* of the flow state scale was the threshold 0.725 and experience 0.910. The higher the score on this scale means a higher level of flow state.

### Data Analysis

In this study, the SPSS 26.0 and Amos 21.0 software were used to analyze the data. First, to verify the validity and reliability of the measurement tools, a confirmatory factor analysis and Cronbach α were conducted with maximum likelihood. Based on these results, we constructed an item parceling for each subfactor and proceed with further analysis. Second, a descriptive statistical analysis was conducted to verify the overall tendency (mean, standard deviation, skewness, and kurtosis) of the collected data and whether normal distribution assumptions were met. Third, a correlation analysis was conducted to verify the relationships between measured variables. Fourth, a confirmatory factor analysis was conducted to determine whether the research model established in this study is appropriate. Subsequently, construct reliability (CR) and average variance extraction (AVE) were calculated to verify the suitability of the research model. Finally, structural equation modeling (SEM) with maximum likelihood was conducted to verify the suitability and research model, and the research model was verified based on the path coefficients estimated. The bootstrapping method was used to examine the significance of the indirect effect (mediated effect).

## Results

### Descriptive Statistics and Correlations

The descriptive statistics analysis and correlation results for each variable are shown in Table 1. Among the subfactors of modeling, skill (*M* = 5.00, *SD* = 1.38) showed the highest tendency, followed by strategy (*M* = 4.44, *SD* = 1.42) and performance (*M* = 4.53, *SD* = 1.45). Subfactors of self-efficacy showed a high tendency in skill (*M* = 7.89, *SD* = 1.56) and performance (*M* = 7.89, *SD* = 1.56). In the subfactors of flow state, threshold (*M* = 4.57, *SD* = 0.91) was followed by experience (*M* = 3.89, *SD* = 1.01). Skewness and kurtosis values did not exceed ±2, satisfying normality. The subfactor of modeling showed positive correlations with the subfactors of self-efficacy and flow state. Also, the relationship between subfactors of self-efficacy and flow state showed positive correlation.

**Table 1:** Result of descriptive statics and correlations.

Variable	*a*	*b*	*c*	*d*	*e*	*f*	*g*	*h*
M: skill	1							
M: strategy	0.788^**^	1						
M: performance	0.732^**^	0.821^**^	1					
E: skill	0.457^**^	0.425^**^	0.338^**^	1				
E: strategy	0.434^**^	0.380^**^	0.354^**^	0.846^**^	1			
E: performance	0.390^**^	0.396^**^	0.355^**^	0.792^**^	0.801^**^	1		
Flow threshold	0.382^**^	0.460^**^	0.434^**^	0.450^**^	0.474^**^	0.452^**^	1	
Flow experience	0.403^**^	0.475^**^	0.434^**^	0.390^**^	0.443^**^	0.422^**^	0.648^**^	1
Mean	5.00	4.44	4.53	7.89	7.75	7.89	4.57	3.89
SD	1.38	1.42	1.45	1.56	1.55	1.50	0.91	1.01
Skewness	−0.815	−0.608	−0.623	−0.469	−0.362	−0.542	−0.581	−0.286
Kurtosis	0.696	0.138	0.163	−0.539	−0.461	−0.252	0.323	−0.066

** *p* < 0.01.

### Verification of Entire Research Model

We estimated the factor loading by applying the maximum likelihood method to verify the construct validity of the entire research model and found that the goodness-of-fit index of the research model was good (*χ*^2^ = 24.341, *df* = 17, TLI = 0.989, CFI = 0.993, RMSEA = 0.048). The standardization coefficient of the subfactors explained from the potential factors was shown to be 0.793 to 0.940, indicating suitable explanatory power. Verifying convergent and discrimination validity through CR, AVE values for subfactors, and coefficient of determination between concepts (modeling, self-efficacy, and flow state), the construct validity of the research model was confirmed (see Table 2).

**Table 2:** CR, AVE values, and coefficient determination of entire research model.

Variables	CR	AVE	Coefficient of determination (*r*^2^)		
			Modeling	Self-efficacy	Flow
Modeling	0.845	0.645	1	–	–
Self-efficacy	0.848	0.650	0.229	1	–
Flow state	0.799	0.665	0.173	0.163	1

### Mediating Effects of Self-Efficacy

We set up a research model that tested the effect of self-efficacy as the mediating variable in the relationship between modeling and flow state through role model of adolescent athletes and conducted structural equation model analysis (SEM) to verify this model. To assess the suitability of the study model, TLI, CFI, and RMSEA were calculated. The research models established in this study were shown to be reasonable, as the TLI, CFI, and RMSEA values all met the goodness-of-fit indices (*χ*^2^ = 24.341, *df* = 17, TLI = 0.989, CFI = 0.993, RMSEA = 0.048). Since the suitability of the study model has been verified, the hypothesis of this study was verified based on the path coefficients estimated through the research model. Each parameter estimate given in Table 3 showed that modeling had a significant positive relationship with self-efficacy and flow state. Also, self-efficacy had a significant positive relationship with flow state.

**Table 3:** Parameter-estimated value of each path way.

Path	Parameter-estimated value
Modeling → flow state	0.244 (0.416)^**^
Modeling → self-efficacy	0.500 (0.479)^**^
Self-efficacy → flow state	0.114 (0.194)^**^

** *p* < 0.01.

Meanwhile, to verify the mediating effects of self-efficacy in the relationship between modeling and flow state, structural equation modeling analysis was conducted. To examine the mediated effects of the research model established in this study, the indirect effects were validated using the boot-strapping analysis proposed by Preacher and Hayes (2008). The statistical significance of the mediating effects was determined based on a 95% confidence interval and the number of samples was set to 1,000 (Cheung and Lau, 2008).

**Figure 1:**
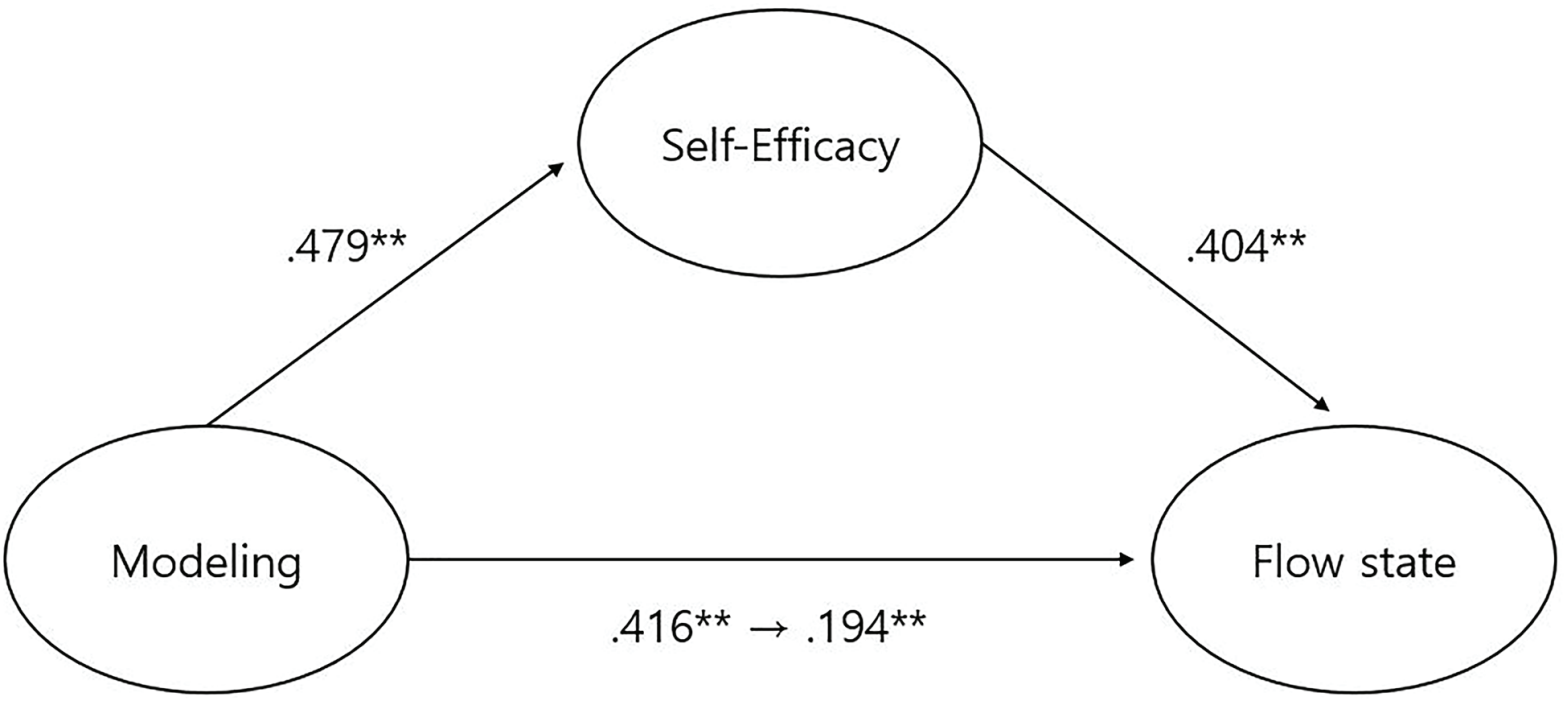
Validation of hypothetical research model.

The direct effects of adolescent athlete modeling on flow state (*β* = 0.416, *B* = 0.244, *p* < 0.01) and self-efficacy (*β* = 0.479, *B* = 0.500, *p* < 0.01) were all significant, and the direct effects of self-efficacy on flow state (*β* = 0.404, *B* = 0.227, *p* < 0.01) were also significant. Furthermore, the indirect effect of adolescent athlete modeling on flow state (*β* = 0.194, *B* = 0.114, *p* < 0.01) was significant, and the partial mediated effect of self-efficacy was significant. That is, adolescent athletes having role models has been shown to have a positive effect on flow state through mediation by self-efficacy (see Table 4; Figure 1).

**Table 4:** Direct and indirect effect.

Pathway	Direct effect	Indirect effect	95% confidence	
			Upper	Lower
Modeling → flow state	0.416^**^	0.000	0.564	0.261
Modeling → self-efficacy	0.479^**^	0.000	0.589	0.345
Self-efficacy → flow state	0.404^**^	0.000	0.554	0.248
Modeling → self-efficacy → flow state	0.000	0.194^**^	0.279	0.114

** *p* < 0.01.

## Discussion

This study was conducted to: (1) examine how the use of modeling through a role model by adolescent athletes affects their self-efficacy and flow state and (2) verify the structural relationship of modeling, self-efficacy, and flow state set as research theories. The main findings are discussed below.

First, modeling through role models was found to improve the self-efficacy of adolescent athletes. Vicarious experience is a form of modeling, and it involves obtaining capability information by observing the performance of others. An individual’s ability affects his or her sense of self-efficacy, but self-efficacy is also affected by observing other people’s performance or achievement. For example, when a player sees other players with similar physique and skill level perform successfully, it convinces them that they too can do the same and increases their self-efficacy (Schunk et al., 1987). In addition, in this study, we examined the effectiveness of modeling through role models by limiting the object of modeling to athletes’ own role models because role models can affect an individual’s behavior, cognition, and emotions. The selection of a positive role model has a positive effect on the self-efficacy of being able to perform like the role model. This finding supports the results of a prior study which found that role modeling improves outcome expectations and stimulates a desire for success in a particular field. Thus, it is suggested that role modeling can be a key source for improving self-efficacy in adolescent athletes.

Second, the self-efficacy of adolescent athletes was found to have a positive effect on the flow state. Although few empirical studies on athletes’ self-efficacy and flow have been conducted, flow is related to sport-confidence, which is very similar to self-efficacy, and self-efficacy is positively correlated with sport-confidence. Comprehensively looking at the results of previous research, it can be concluded that a high level of self-efficacy improves expectations of success and self-confidence and that improved self-efficacy can serve as a leading variable to promote the flow state during a competition.

Third, in this study, modeling through a role model had a positive effect on the flow state. Although there are not many prior studies showing that modeling affects flow, Kwon and Shin (2020) reported that observational learning (e.g., modeling) had a positive impact on the flow and noted that subprocessing of observational learning may theoretically affect flow. That is, the act of selecting role models and then observing the selected models to mimic specific skills or behaviors can be linked to a clear focus on goals and tasks that is cited as the essential element for the expression of flow experience. Meanwhile, Bakker (2005) reported that the more flow experience a music teacher has, the more frequent the flow experience of students, and they said that just as emotions are contagious, flow can also be contagious. Thus, in the crossover process of such emotions, students can make unconscious or conscious imitation that can be achieved through happy and cheerful emotions from teacher. Although this research was not done in sport context, the results of the research can provide a mechanism to infer the relationship between modeling and flow state.

Finally, the relationship between the use of role modeling by adolescent athletes and the flow state was verified by establishing a structural model in which self-efficacy is mediated. The mediating effect of self-efficacy was confirmed. These structural relationships can be interpreted through the results of the preceding studies discussed earlier. A modeling method in which individuals select their own role models and observe and emulate them enhances self-efficacy and builds a belief in the individual that they can achieve what the role model has achieved, raising expectations of success and motivating them to work hard towards their goals. Essentially, role models provide a strong motivation to achieve clearly defined goals, which is required for the flow state to arise.

### Limitations and Future Research

There are some limitations and recommendations which should be considered for future research. First, in this study, the gender ratio was set based on the data from the Korean Sport & Olympic Committee. Therefore, subsequent studies need to be expanded to a variety of disciplines, with the appropriate number of samples for each discipline. Further research needs to be conducted to generalize the results by adding female athletes and various types of sports and sports events.

Second, modeling through role models does not always increase self-efficacy and flow state. Who the role model is and how similar he/she is to oneself is a variable that can affect the effectiveness of role modeling and should be taken into consideration. For role modeling to improve self-efficacy and performance, the role model needs to be similar to the individual. George et al. (1992) found that higher similarity with the role model have a more positive effect on self-efficacy and performance in comparison with low similarity. Although that study was conducted on a typical population, it can be applied to athletes. In this study, similarities with role models were not set as background variables, but future studies must consider the effect of similarity of between athletes and their role models in terms of gender, age, motor skills, etc.

Third, the ultimate focus of sports psychology is how to achieve peak performance. In this study, the effectiveness of adolescent athletes’ peak performance was verified by experience of flow state. Csikszentmihalyi (1985) noted that if the athlete was performing at the peak performance, the athlete can be considered to be in a state of flow. However, the state of flow does not necessarily bring about the peak performance because the peak performance is differently recognized and interpretable by each athlete. Therefore, if future studies select sports in which performance can be measured using objective indicators, such as golf and track and field, and compare it with performance produced by the state of flow, we will get more diverse and reasonable results.

## Data Availability Statement

The original contributions presented in the study are included in the article/supplementary material, further inquiries can be directed to the corresponding author.

## Ethics Statement

The studies involving human participants were reviewed and approved by Institutional Review Board of the Seoul National University. Written informed consent to participate in this study was provided by the participants’ legal guardian/next of kin.

## Author Contributions

SK contributed to the literature review and supervision. JA analyzed and interpreted the data. SL wrote the original draft and edited the manuscript. All authors have read and agreed to the published version of the manuscript.

### Conflict of Interest

The authors declare that the research was conducted in the absence of any commercial or financial relationships that could be construed as a potential conflict of interest.
