# Supplementary material for: The Effect of Modeling on Self-Efficacy and Flow State of Adolescent Athletes Through Role Models
Source: Front Psychol. 2021 Jun 14;12:661557. doi: 10.3389/fpsyg.2021.661557 (PMC8236606; doi:10.3389/fpsyg.2021.661557)
Supplement: Supplementary file 1 [file Data_Sheet_1.docx]

Appendix 1

| Functions of Observational Learning Items | |
| --- | --- |
| 1 | I use observational learning (through my role models) to understand how to get psyched up. |
| 2 | I use observational learning (through my role models) to understand what it takes to be mentally tough. |
| 3 | I use observational learning (through my role models) to learn how to cope with anxiety. |
| 4 | I use observational learning (through my role models) to know how to respond to the excitement associated with performing. |
| 5 | I use observational learning (through my role models) to learn how to be focused during a challenging situation. |
| 6 | I use observational learning (through my role models) to assist me in staying positive in tough situations. |
| 7 | I use observational learning (through my role models) to help me properly perform a physical skill. |
| 8 | I use observational learning (through my role models) to improve my skills. |
| 9 | I use observational learning (through my role models) to change how I perform a skill. |
| 10 | I use observational learning (through my role models) to understand how to perfectly perform a skill. |
| 11 | I use observational learning (through my role models) to help me learn new skills. |
| 12 | I use observational learning (through my role models) to help me fine tune my skills. |
| 13 | I use observational learning (through my role models) to make up new plans/strategies in my head. |
| 14 | I use observational learning (through my role models) to form alternative plans or strategies. |
| 15 | I use observational learning (through my role models) to develop game plans and routines. |
| 16 | I use observational learning (through my role models) to determine how a strategy will work in an event/game. |
| 17 | I use observational learning (through my role models) to help me improve my game/event strategies. |

Appendix 2

| Self-Efficacy Items | |
| --- | --- |
| 1 | I am confident I can learn the skills necessary to play this sport. |
| 2 | I am confident I can Improve the skills I have already acquired. |
| 3 | I am confident I can master the skills required in this sport. |
| 4 | I am confident I can master the strategies required in this sport. |
| 5 | I am confident I can learn the strategies necessary to play this sport. |
| 6 | I am confident I can improve the strategies I have already acquired. |
| 7 | I am confident I can stay motivated to play this sport. |
| 8 | I am confident I can stay focused when playing this sport. |
| 9 | I am confident I can stay positive when playing this sport. |

Appendix 3

| Flow State Items | |
| --- | --- |
| 1 | I completely focus on the movement I'm doing. |
| 2 | I totally flow in the game. |
| 3 | I only think about my record or movement. |
| 4 | When I’m in a state of flow, all my focus is on special movements. |
| 5 | Everything is going my way. |
| 6 | I feel like I'm one with the exercise equipment. |
| 7 | I feel like my body is moving the way I think. |
| 8 | I can lead the game as I want. |
| 9 | I feel that movements take place automatically. |
| 10 | I feel the movements that were difficult for me to do are automatically performed. |
